# Total neoadjuvant therapy with short-course radiotherapy Versus long-course neoadjuvant chemoradiotherapy in Locally Advanced Rectal cancer, Korean trial (TV-LARK trial): study protocol of a multicentre randomized controlled trial

**DOI:** 10.1186/s12885-023-11177-7

**Published:** 2023-08-08

**Authors:** Min Jung Kim, Dae Won Lee, Hyun-Cheol Kang, Ji Won Park, Seung-Bum Ryoo, Sae-Won Han, Kyung Su Kim, Eui Kyu Chie, Jae Hwan Oh, Woon Kyung Jeong, Byoung Hyuck Kim, Eun Mi Nam, Seung-Yong Jeong

**Affiliations:** 1 Department of Surgery, Seoul National University College of Medicine, 101 Daehak-Ro, Jongno-Gu, Seoul, 03080 Korea; 2 Cancer Research Institute, Seoul National University, Seoul, Korea; 3 Department of Internal Medicine, Seoul National University College of Medicine, Seoul, Korea; 4 Department of Radiation Oncology, Seoul National University College of Medicine, Seoul, Korea; 5 Cancer for Colorectal Cancer, National Cancer Center, Goyang, Korea; 6 Department of Surgery, Keimyung University Dongsan Medical Center, Keimyung University School of Medicine, Daegu, Korea; 7 Department of Radiation Oncology, Seoul Metropolitan Government Seoul National University Boramae Medical Center, Seoul, Korea; 8 Department of Internal Medicine, Ewha Womans University College of Medicine, Seoul, Korea

**Keywords:** Total neoadjuvant therapy, Rectal cancer, Chemoradiotherapy, Radiotherapy, Chemotherapy, Pathologic complete response

## Abstract

**Background:**

For locally advanced rectal cancer (LARC), total neoadjuvant therapy (TNT) may enhance tumour response, reduce recurrence, and improve patient compliance compared to upfront surgery. Recent studies have shown that chemoradiotherapy (CRT) followed by consolidation chemotherapy leads to higher rate of pathologic complete response (pCR) than induction chemotherapy followed by CRT. However, an optimal TNT regimen that maximise the pCR rate and minimise toxicity has not been established. Therefore, the aim of this trial was to investigate whether preoperative short-course radiotherapy followed by chemotherapy with four cycles of CAPOX can double the pCR rate compared to a standard schedule of long-course preoperative CRT in patients with LARC.

**Methods:**

This is a multi-centre, prospective, open label, randomised controlled trial. Patients with clinical primary tumour stage 3 and higher or regional node-involved rectal cancer located within 10 cm from the anal verge were randomly assigned equally to short-course radiotherapy (25 Gy in 5 fractions over 1 week) followed by four cycles of CAPOX (intravenous oxaliplatin [130 mg/m^2^, once a day] on day 1 and capecitabine [1,000 mg/m^2^, twice a day] from days 1 to 14) (TNT) or CRT (50.4 Gy in 28 fractions over 5 weeks, concurrently with concomitant oral capecitabine 825 mg/m^2^ twice a day). After preoperative treatment, total mesorectal excision was performed 2–4 weeks in the TNT group and 6–10 weeks in the CRT group, followed by optional additional adjuvant chemotherapy. The primary endpoint is the pCR rate, and secondary endpoints include disease-related treatment failure, quality of life, and cost-effectiveness. Assuming a pCR rate of 28% and 15% in the TNT and CRT groups, respectively, and one-side alpha error rate of 0.025 and power of 80%, 348 patients will be enrolled considering 10% dropout rate.

**Discussion:**

The TV-LARK trial will evaluate the superiority of employed TNT regimen against the standard CRT regimen for patients with LARC. We aimed to identify a TNT regimen that will improve the pCR rate and decrease systemic recurrence in these patients.

**Trial registration:**

Cris.nih.go.kr ID: KCT0007169 (April 08, 2022). The posted information will be updated as needed to reflect the protocol amendments and study progress.

**Supplementary Information:**

The online version contains supplementary material available at 10.1186/s12885-023-11177-7.

## Background

Neoadjuvant long-course chemoradiotherapy (CRT), followed by total mesorectal excision (TME) and adjuvant chemotherapy, has been the standard treatment for patients with locally advanced rectal cancer (LARC) [1–4]. This multimodal treatment has evolved to its current regimen through trials that have proven its effectiveness. In Dutch and Swedish trials, radiotherapy plus surgery reduced local recurrence and significantly improved overall and cancer-specific survival [3, 5]. Addition of chemotherapy to radiotherapy resulted in less advanced pathological tumour stages and lower incidences of local recurrences than radiotherapy alone [2, 6]. In German trial, preoperative radiotherapy reduced local recurrence and toxicity compared to postoperative radiotherapy alone [4, 7]. The Lyon R90-01 and GRECCAR-6 trials demonstrated that delaying surgery for 6 to 8 weeks increased tumour downstaging [8, 9]. The recent ADORE trial confirmed that adjuvant FOLFOX improved disease-free survival (DFS) after neoadjuvant CRT [10]. Thus, preoperative CRT followed by delayed TME with adjuvant chemotherapy has been the standard treatment for LARC for decades, and the local recurrence in major centres has improved to as low as 5% to 8% [11].

However, the rate of distant metastasis surpassed local recurrence in patients with rectal cancer under modern treatment regimens [2]. In a prospective trial, the 10-year cumulative incidence of distant metastasis was approximately 29% in both preoperative and postoperative radiotherapy groups, whereas the 10-year cumulative incidence of local recurrence rate was 6.8% and 9.4%, respectively [7]. Two randomised controlled trials demonstrated that adjuvant fluorouracil and leucovorin (FL) chemotherapy could not prevent distant metastasis in rectal cancer patients undergoing preoperative CRT and surgery [12, 13]. The low compliance to the planned adjuvant chemotherapy is one of the reasons for the high rate of distant metastasis because only about half of patients finish adjuvant chemotherapy [2, 12, 14]. Moreover, in the setting of conventionally fractionated preoperative CRT, patients will receive systemic chemotherapy 15 to 19 weeks after starting treatment. Therefore, researchers have attempted to reduce the duration of radiotherapy and move the systemic chemotherapy preoperatively as consolidation or induction chemotherapy [15–17].

Total neoadjuvant therapy (TNT) for rectal cancer is a therapeutic strategy that incorporates chemotherapy with CRT before surgery to eradicate occult micro-metastasis and increase the pathologic complete response (pCR) rate. In the STELLAR trial, four cycles of CAPOX were administered with short-course RT before surgery. The TNT group showed improved overall survival compared with the conventional CRT group, and the total rate of pCR and sustained cCR in TNT group was 21.8%, which was significantly higher than in the CRT group's pCR of 12.3% (*p* = 0.002) [18]. The RAPIDO trial performed short-course RT and six cycles of CAPOX for locally advanced rectal cancer with high-risk features. The 3-year disease-related treatment failure rate was 23.7% (19.8–27.6) vs. 30.4% (26.1–34.6), and the pCR rate was 28% vs. 14% (*p* < 0.0001) [19]. The UNICANCER-PRODIGE 23 trial in France used FOLFIRINOX #6 and long-course CRT as intensified neoadjuvant chemotherapy for cT3 or cT4M0 LARC [20]. The results showed that compared to the standard care group, the 3-year DFS was improved significantly (HR 0.69, *p* = 0.034), and the pCR rate was 28% vs. 12% (*p* < 0.0001).

Improving the pCR rate is important for organ preservation in patients with rectal cancer. In the Memorial Sloan-Kettering Cancer Centre, researchers suggested that TNT can facilitate non-operative treatment, giving a higher pCR rate from their retrospective cohort analysis [21]. In addition, by comparing induction chemotherapy and consolidation chemotherapy, they suggested that consolidation chemotherapy followed by CRT could achieve more rectal preservation than induction chemotherapy by enhancing the sustained clinical CR rate [22].

However, the TNT regimen for the standard-risk LARC has not been standardised to maximise its effectiveness in increasing pCR and decreasing distant metastasis while minimising treatment-related toxicity. Two randomised controlled trials confirmed comparable local control and DFS between short-course radiotherapy and long-course CRT [14, 23]. Three months of CAPOX treatment was as effective as 6 months treatment in stage III colon cancer, except for T4 or N2 cancers in the IDEA study [24]. Therefore, this manuscript aimed to report the protocol for the prospective randomized controlled trial that will compare short-course radiotherapy followed by four cycles of CAPOX with conventionally fractionated preoperative CRT for LARC.

## Methods

### Design

**Fig. 1:**
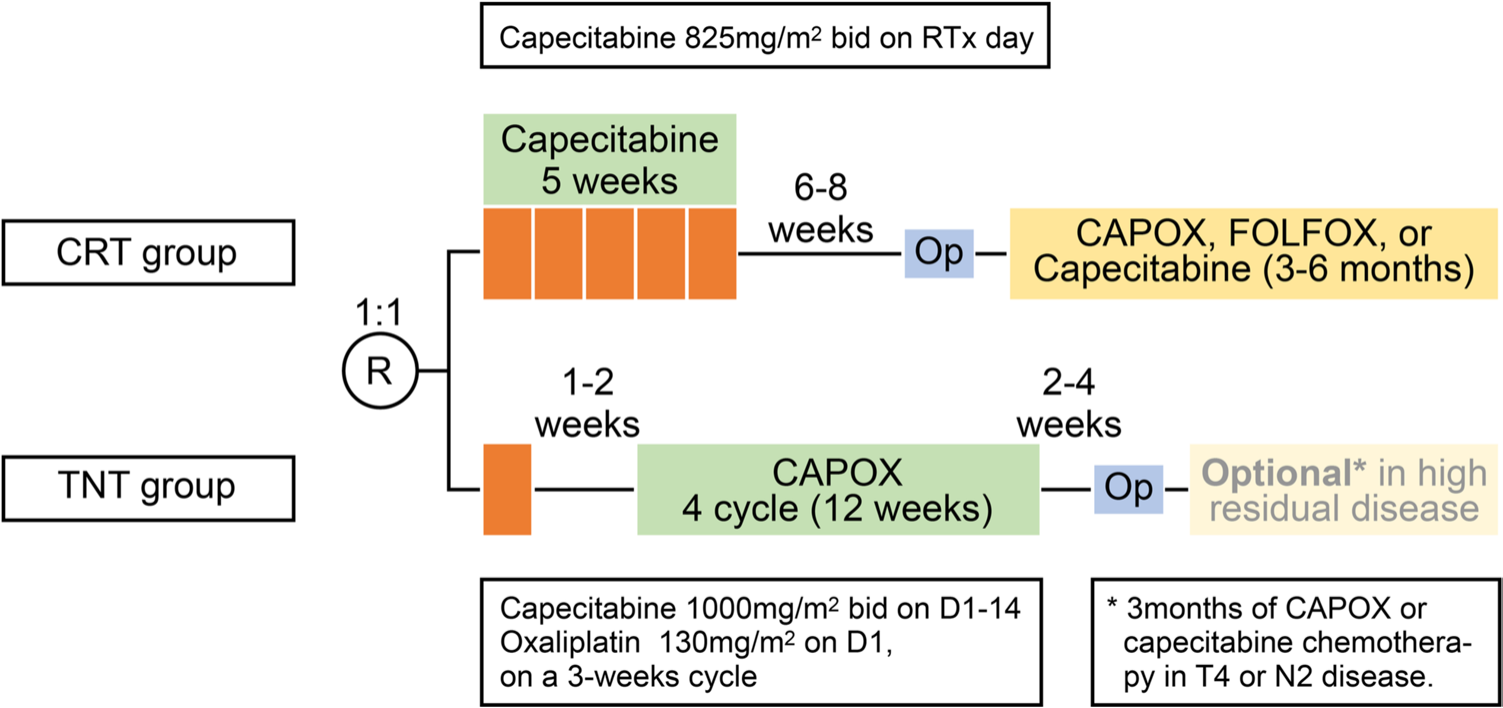
Study flow. Orange bar represents radiotherapy per one week. Abbreviations: *RTx* Radiotherapy, *CRT* Chemoradiotherapy, *TNT* Total neoadjuvant therapy, *Op* Operation

The TV-LARK trial is a multicentre randomised phase III superiority trial initiated by the rectal cancer research group in the Seoul National University Hospital. A list of all participating centres has been added as a Supplementary file 1. Eligible patients were randomly assigned to receive either short-course radiotherapy and neoadjuvant chemotherapy followed by surgery (intervention; arm A) or neoadjuvant CRT followed by surgery and adjuvant chemotherapy (comparator; arm B) (Fig. 1). Randomisation in a 1:1 ratio was performed centrally using a web-based system, with stratification according to clinical T (cT2-3 vs. cT4) and N (cN0 vs. cN +) stage.

### Study population

Patients are eligible if they have histologically confirmed resectable stage II or III rectal adenocarcinoma without distant metastases, including para-aortic lymph nodes (LNs) and common and external iliac LN metastases. Resectability was assessed by a multiphase computed tomography (CT) scan within four weeks before randomisation. The inclusion criteria were a tumour located ≤ 10 cm from the anal verge, patient aged 19–80 years, Eastern Cooperative Oncology Group (ECOG) performance status scale ≤ 1, American Society of Anesthesiologists (ASA) class I or II, no history of any other systemic treatment or radiotherapy for rectal cancer, no history of intraoperative radiotherapy, adequate bone marrow function (i.e. absolute neutrophils ≥ 1,500/mm^3^; platelets ≥ 75,000/mm^3^), adequate liver function (bilirubin ≤ 2.0 fold of upper limit of normal, liver function enzymes level (aspartate aminotransferase, alanine aminotransferase) ≤ 2.5 fold of upper limit of normal), and adequate renal function (creatinine ≤ 1.5 fold of upper limit of normal or renal filtrate rate (Ccr, calculated using Cockcroft formula) ≥ 50 ml/min. Patients who can comply with the study protocol for the duration of the study, understand the study process and treatment plan, and have signed the informed consent form will be included.

The exclusion criteria were other malignancies within five years except for cured superficial skin cancer or cervical carcinoma in situ; prior treatment for rectal cancer; history of organ transplant requiring immunosuppressive therapy; uncontrolled epilepsy or psychosis; hypersensitivity to fluoropyrimidine agents, platinum, leucovorin, capecitabine, or confirmed dihydropyridine dehydrogenase deficiency; and genetic problems such as galactose intolerance, Lapp lactase deficiency, or glucose-galactose malabsorption. Patients who were required to continue concomitant treatment and were expected to receive oxaliplatin, flucytosine, phenytoin, or warfarin were also excluded. Furthermore, patients were ineligible in cases of National Cancer Institute Common Toxicity Criteria grade 1 or higher peripheral neuropathy, uncontrolled or severe cardiovascular disease, infection, and pregnancy.

### Treatment

#### Arm A: total neoadjuvant short-course radiotherapy followed by CAPOX (TNT group)

The treatment in arm A started with short-course radiotherapy (25 Gy in 5 fractions during a week). A topical simultaneous integrated boost was permitted at the investigator’s discretion. The contouring of the radiotherapy plan was the same as that of the control group. Neoadjuvant chemotherapy will be given within two weeks after the last radiotherapy fraction but should be within at least three weeks. Chemotherapy consisted of four cycles of CAPOX (capecitabine 1,000 mg/m^2^ orally twice daily on days 1 − 14, oxaliplatin 130 mg/m^2^ intravenously on day 1, and a chemotherapy-free interval between days 15 − and 21). The dose was adjusted based on the maximum graded toxicity within the previous cycle. Within a week after the last day of neoadjuvant chemotherapy, restaging CT scan, MRI, and sigmoidoscopy were performed and, when appropriate, followed by surgery with curative intent. Surgery is planned 2 to 4 weeks after neoadjuvant chemotherapy. Adjuvant chemotherapy is not scheduled but can be administered at the investigator’s discretion according to the pathologic tumour response.

#### Arm B: neoadjuvant chemoradiotherapy and adjuvant chemotherapy (standard of care group)

Treatment in arm B starts with radiotherapy in 28 daily fractions of 1.8 Gy up to 50.4 Gy or 25 fractions up to 50.0 Gy with concomitant oral capecitabine 825 mg/m^2^ twice a day. In both groups, the clinical target volume (CTV) 1 was defined as an area extending 1.5 cm or more to the distal and proximal direction of the tumour in the rectum bordered by the internal iliac and presacral LNs and mesorectal fascia on CT images in the prone position. CTV 2 was defined as CTV 1 plus enlarged LN areas. Planning target volume (PRV) 1 and PTV 2 will cover 0.8 − 1 cm from CTV 1 and CTV 2, respectively. The radiation dose was delivered to 95% of the PTV, and there was no area where the radiation dose is given exceeding 10% or more than the prescribed. A three-beam technique was used, and the treating physician and hospital policy permitted intensity-modulated radiotherapy. Six weeks after the last day of CRT, a restaging examination similar to that of the TNT group was performed. Surgery was planned six − to eight weeks after the last day of CRT. Adjuvant chemotherapy was administered within 3 − 8 weeks after surgery. Capecitabine alone or CAPOX was administered for 3–6 months (4–8 cycles) at the investigator’s discretion. Capecitabine alone was administered at 1,250 mg/m^2^ twice a day on days 1 − 14, with three weeks per cycle. CAPOX was administered as oxaliplatin 130 mg/m^2^ on day 1 and capecitabine 1,250 mg/m^2^ twice a day on days 1 − 14.

### Surgery: both groups

Surgery is performed according to TME principles. The rectum was dissected along the holy plane of the mesorectum. The lateral LN is additionally dissected if metastasis is suspected on preoperative MRI. Surgical approaches include laparotomy, laparoscopy, robotics, and transanal TME. Rectal preservation strategies, such as local excision or watch-and-wait, are not considered in this protocol. Postoperative complications were defined according to the Clavien-Dindo classification and recorded until 30 days after surgery. If neoadjuvant chemotherapy or radiotherapy is discontinued because of toxicity or in cases of local progression, patients will still proceed with the surgery. Patients who develop distant metastasis or unresectable disease during neoadjuvant therapy at restaging on the other hand will proceed with chemotherapy.

### Outcomes

The primary endpoint was pCR, which will be evaluated by independent pathologists at each institution. Secondary endpoints included disease-related treatment failure (first occurrence of locoregional failure, new primary colorectal cancer, distant metastasis, or treatment-related death) [19], quality of life (patient-reported outcomes), and cost-effectiveness analysis. The exploratory endpoint is circulating tumour DNA (ctDNA), which will be measured to confirm whether detecting a treatment response, relapse, or relapse at an earlier phase is possible.

### Follow up

After treatment, follow-up will be conducted every six months for five years. Physical exam, tumour markers (carcinoembryonic antigen [CEA] and carbohydrate antigen 19–9 [CA19-9]), and chest and abdomen CT scans will be taken every six months until disease recurrence or up to a maximum of 5 years after the end of treatment without recurrence. Total colonoscopy was planned at 12, 36, and 60 months postoperatively. Rectal MRI and PET CT scans are allowed on indication to detect or confirm recurrence.

### Quality of life and functional outcomes

The quality of life and functional outcomes were assessed using patient-reported questionnaires at multiple time points throughout the treatment and during follow-up. The questionnaires included two European Organization for Research and Treatment of Cancer (EORTC) questionnaires: the quality-of-life questionnaire for patients with cancer (QLQ-C30) and the quality-of-life questionnaire for patients with colorectal cancer (QLQ-CR29). Postoperative bowel function was measured using the low anterior resection syndrome (LARS) score. These questionnaires are available in Korea and have been validated in previous studies [25–27].

### Sample size calculation and statistical analysis

The study hypothesis was that when the pCR rate of the conventional CRT group (arm B) was assumed to be 15%, the pCR rate will be increased to 28% in the TNT group (arm A). With a one-sided alpha of 0.025 and a power of 80%, each group needed 156 participants to prove the hypothesis. Considering the 10% of dropout rate, a total of 348 patients will be required.

### Data collection and management

The web-based software iCReaT version 2.0 by the National Institute of Health of the Korean Disease Control and Prevention Agency was used for randomisation, data collection, and central data monitoring and management. Data management was coordinated by HERINGS, Co. Ltd., and clinical research coordinators of each institution collected data. Data were entered following a predefined and standardised protocol, providing guidelines for missing values. Data managers were trained using the iCReaT software and electronic case report form before data entry.

### Monitoring

Qualified and independent monitoring will be performed periodically throughout the trial by HERINGS and the institutional IRB. The participating centres will be visited to randomly check the compliance with the protocol and enrolment criteria, proper treatment implementation, query review, data verification, and adverse event reports. Adverse events were graded using the Common Terminology Criteria for Adverse Events (CTCAE) version 5.0 [28].

## Discussion

We describe the protocol of the TV-LARK trial, a multicentre randomised phase III trial supported and funded by the National Evidence-based Healthcare Collaborating Agency (NECA) in South Korea. The trial was designed to prove the efficacy of short-course radiotherapy followed by TNT for patients with LARC. This study was based on previous studies regarding TNT in rectal cancer. If TV-LARK trial demonstrates superior pCR for patients receiving short-course radiotherapy with four cycles of CAPOX to those receiving long-course CRT with adjuvant chemotherapy, this protocol could be accepted as the neoadjuvant treatment of choice for patients with LARC.

Studies have demonstrated that TNT for LARC elevated the pCR rate, reduced distant metastasis, and improved patient compliance to chemotherapy compared with long-course neoadjuvant CRT. A meta-analysis of seven studies, including randomised controlled trials or cohort studies, showed that TNT was associated with improved pCR and had a potential survival advantage compared with the standard CRT strategy in LARC [29]. The pooled prevalence of pCR was 29.9% in the TNT group and 14.9% in the standard CRT group.

### Supplementary Information

**Additional file 1. **

## Data Availability

The trial is registered at cris.nih.go.kr (KCT0007169).
