# Supplementary material for: Total neoadjuvant therapy with short-course radiotherapy Versus long-course neoadjuvant chemoradiotherapy in Locally Advanced Rectal cancer, Korean trial (TV-LARK trial): study protocol of a multicentre randomized controlled trial
Source: BMC Cancer. 2023 Aug 8;23:734. doi: 10.1186/s12885-023-11177-7 (PMC10408179; doi:10.1186/s12885-023-11177-7)
Supplement: Supplementary file 1 — Additional file 1. [file 12885_2023_11177_MOESM1_ESM.docx]

**Supplementary file TV-LARK trial**

**List of participating centers:**

Ewha Womans University Seoul Hospital, Seoul, Korea

Keimyung University Dongsan Medical Center, Daegu, Korea

National Cancer Center, Goyang, Korea

Samsung Medical Center, Seoul, Korea

Seoul Asan Medical Center, Seoul, Korea

Seoul Metropolitan Government Seoul National University Boramae Medical Center, Seoul, Korea

Seoul National University Hospital, Seoul, Korea

**List of affiliated centers:**

Ewha Womans University Seoul Hospital, Seoul, Korea

Keimyung University Dongsan Medical Center, Daegu, Korea

National Cancer Center, Goyang, Korea

Seoul Metropolitan Government Seoul National University Boramae Medical Center, Seoul, Korea

Seoul National University Hospital, Seoul, Korea
